# The impact of global health opportunities on residency selection

**DOI:** 10.1186/s12909-021-02795-5

**Published:** 2021-07-15

**Authors:** Caitlin Kaeppler, Peter Holmberg, Reena P. Tam, Kelsey Porada, Shanna D. Stryker, Kate Conway, Charles Schubert, On behalf of the Medical Student Global Health Study Group, Stacey Chamberlain, Carmen Cobb, James H. Conway, Elizabeth Groothuis, Ebba Hjertstedt, Suet Kam Lam, Megan McHenry, Theresa Nguyen

**Affiliations:** 1 Department of Pediatrics, Medical College of Wisconsin, Milwaukee, WI USA; 2 Children’s Wisconsin Corporate Center, 999 N 92nd St, Suite 560, Wauwatosa, WI 53226 USA; 3 Department of Pediatrics, Mayo Clinic, Rochester, MN USA; 4 Department of Pediatrics, University of Utah School of Medicine, Salt Lake City, UT USA; 5 Clinical Research Coordinator, Medical College of Wisconsin, Milwaukee, WI USA; 6 Department of Family/Community Medicine, University of Cincinnati College of Medicine, Cincinnati, OH USA; 7 Department of Family Medicine, Boonshoft School of Medicine, Wright State University, Dayton, OH USA; 8 Departments of Family/Community Medicine and Pediatrics, University of Cincinnati College of Medicine, Cincinnati, OH USA

**Keywords:** Global health, Residency selection, Residency curriculum, Underserved populations

## Abstract

**Background:**

An increasing number of medical trainees across specialties desire and expect Global Health (GH) experiences during training. It is useful for residency programs to know the impact that offering GH opportunities has on resident recruitment. The study objectives were to explore the importance of GH opportunities in residency selection among fourth-year medical students, examine the relationship between interest in GH and career plans, and describe students’ perspectives on prior GH experiences.

**Methods:**

The authors administered an electronic survey to all fourth-year medical students attending 12 different US institutions in February 2020. Data from the ten schools who were able to comply with the survey distribution methodology and with response rates above 25% were analyzed using descriptive statistics and Pearson’s correlation.

**Results:**

A total of 707 fourth-year medical students from the included schools completed the survey out of 1554 possible students (46% response rate). One third of respondents ranked the presence of GH experiences in residency as moderately or very important and 26% felt that the presence of a formal GH curriculum was at least moderately important, with variation noted among specialties. After training, 65% of students envision practicing internationally in some capacity. A desire to care for underserved patients in their careers was significantly correlated with an interest in GH experiences during residency.

**Conclusions:**

The opportunity to be involved in GH experiences during training can be an important factor for many medical students when considering residency choice, and the availability of these opportunities may be a valuable recruitment tool. Students valuing GH opportunities during residency are more interested in working with underserved populations in their future careers.

## Background

Global Health (GH) education and experiences are becoming an increasingly popular, and ever more important aspect of medical training [1]. As our world becomes more interconnected and international travel increases, physicians are expected to have a global understanding of health in order to provide optimal care to their patients. This includes knowledge of common and rarer infectious diseases, the health disparities faced by immigrants and refugees, cultural sensitivity and awareness, and skills at effectively working with medical interpreters, allied health professionals, and humanitarian agencies.

GH electives in medical training have many educational benefits, including increased appreciation of cost barriers associated with patient care, increased perceived utility of the history and physical examination, improved resource allocation and decreased utilization of diagnostic tests, more positive attitudes towards public health interventions, increased awareness of the social determinants of health, and enhanced cultural sensitivity [1, 2]. Trainees gain valuable clinical experience by encountering a wide array of new pathologies and learning to provide high quality medical care with limited resources. They benefit from learning new approaches to common ailments and seeing diseases in more advanced stages than they typically present in the United States (US) [3]. GH electives provide an opportunity for personal and professional development, through working with diverse cultures and patient populations trainees may not otherwise encounter [4]. The skills learned from GH electives advance trainees’ competence as domestic physicians, with particular improvement in the ability to serve immigrant and vulnerable populations in the US [5]. GH education during medical training helps to prepare physicians to care for the growing immigrant and refugee population in the US. A recent survey by Butteris et al demonstrated that not all pediatricians feel comfortable caring for these groups of children, though respondents with international experience were more likely to report high levels of comfort caring for refugees, immigrants, and children traveling internationally [6].

Medical trainees are increasingly seeking out GH experiences [7]. In the 2008 American Academy of Pediatrics survey of graduating pediatric residents, 21% reported participating in GH training during residency [1]. In order to meet this interest, a growing number of residency programs are offering GH education and experiences, though there is substantial variation among specialties. In general, there are fewer GH opportunities in surgical residencies as compared to medical ones [8]. In a 2006–2007 survey of 106 pediatric residency programs in the US, Puerto Rico, and the Caribbean, more than half of programs reported having a GH elective available in the preceding year, and nearly half included formal GH training within the established residency curriculum [9]. More recent data show a substantial increase in the total number, but similar overall percentage (58%) of pediatric programs that offer international health electives [10]. Further, Haq et al found that about one in four pediatric residency programs now offer a dedicated GH track [11]. While the availability of GH education and experiences is increasing, the quality of GH curriculum varies. However, recent publications have begun to put forth consensus recommendations and minimum standards for GH tracks [12, 13]. These guidelines suggest that educational offerings include a longitudinal curriculum, a GH rotation with international or domestic underserved experiences, predeparture preparation, preceptorship during GH electives, post-return debriefing, and scholarly output [11, 12].

In addition to the associated educational and recruitment benefits, GH opportunities during medical training likely also influence career choice. Residents who participate in GH electives are more likely to pursue careers in resource-limited settings locally or abroad, in academics, and in public service [2]. Participating in a GH elective during residency has been shown to be strongly associated with post-residency work in GH or with underserved (usually immigrant) populations [5].

Previous studies have focused on data from residents and residency programs, but none have surveyed fourth-year medical students in the process of applying for residency. Given the importance of GH training and potential benefits in recruitment, we sought to explore fourth-year medical students’ perspectives on (1) influence of GH opportunities on residency choice, (2) future career plans related to GH, and (3) prior GH experiences.

## Methods

An electronic Qualtrics survey was developed by members of the Midwest Consortium of Global Child Health Educators, of which many authors are members. Face validity was determined by the authors, which includes interdisciplinary undergraduate and graduate medical educators at five US midwestern colleges of medicine with active GH work. To assess the feasibility of our study and overall interest in GH experiences during residency training, a pilot study was performed at five of the included institutions in March 2019, yielding 212 responses (32% response rate). The initial study confirmed our suspicions that availability of GH opportunities can be a factor in medical students’ choice of residency. Over one-third of respondents found the availability of GH experiences in residency to be moderately or very important. After presenting the results of the pilot study to the Midwest Consortium of Global Child Health Educators in Fall 2019, the study group was expanded to include a total of 12 medical schools across the US. GH educators in the Midwest Consortium of Global Child Health Educators and the Midwest Universities for Global Health were invited to have their institution participate in the study. The initial survey was modified, and the study design was refined; notable changes included broadening the definition of GH elective rotations in medical school to include work with local underserved, refugee, and immigrant communities, and omitting the words “global health” from the invitation email in order to decrease chance of response bias. Institutional review boards from each institution approved or exempted the project. An informational consent letter was provided to each participant at the start of the survey that invited them to participate in a brief research survey regarding their perspective on the presence of certain curricula in medical school and residency. Surveys were administered via an emailed internet link to all fourth-year medical students at 12 US medical schools in February 2020. The survey was administered in the month prior to residency Match Day to help minimize recall bias. The survey consisted of 14 questions utilizing Likert scales, multiple-choice, and multiselect questions (Table 1). The questions evaluated students’ perceived importance of GH education during residency, level of interest in incorporating GH in their future careers, and prior GH experience. There was no compensation provided to complete the survey.

**Table 1:** Survey Items

Domains and Survey Items	Response Choices
**Influence on Residency Choice**	
When you are selecting your residency program, how important to you is the potential for Global Health opportunities at the institution?	Very important, Moderately important, Slightly important, Not important
*IF ≥ SLIGHTLY IMPORTANT:* What are your reasons for desiring Global Health opportunities in residency? (Check all that apply)	Interest in working internationally for at least part of my career Opportunity to learn about new cultures Opportunity to work in low-resource settings Opportunity to work on foreign language skills Opportunity to travel Research interests abroad Sense that a Global Health experience will improve my compassion and empathy Desire to learn about medical diagnoses not common in the U.S. Other reason (please specify)
How important is it to you that your residency program has a Global Health curriculum (track, certificate, or group)?	Very important, Moderately important, Slightly important, Not important
I will only consider residencies that offer Global Health opportunities.	Strongly agree, Somewhat agree, Neither agree nor disagree, Somewhat disagree, Strongly disagree
**Career Plans**	
In an ideal world, the location of my future practice would be:	Majority internationally Majority in the U.S. with a significant portion internationally Primarily in the U.S. with minor portion internationally Completely in the U.S.
When I have completed medical training, I hope to work with:	Only underserved populations Primarily underserved populations A minor portion of my patients who are underserved No specific interest in working with underserved populations
In what field of medicine did you apply for residency?	Text entry
**Prior GH Experience**	
Did you participate in an elective rotation in a low-resource setting (e.g. internationally or Indian Health Services)? What type of Global Health elective did you participate in? (Check all that apply)	Yes or No International Local low-resource setting Indian Health Services US-Mexico border Refugees Immigrants Other
*IF YES:* How did this experience affect your likeliness to seek further global health experiences during residency?	Much more likely, Slightly more likely, Neutral, Slightly less likely, Much less likely
*IF NO:* What were the reasons that you did not? (Check all that apply)	I did not have interest in doing so Cost Not available at my institution Inability to secure time for the rotation Family obligations Concern for my own health Concern for own safety Other (please specify)
In medical school, I have had dedicated class time on how to provide culturally sensitive care to immigrants and refugees.	No dedicated time Some dedicated time but not enough Adequate dedicated time Frequent dedicated time
In medical school, how often did you have the opportunity to provide clinical care to immigrants and refugees?	Never Some but not enough Adequate encounters Frequent encounters
I feel confident in my ability to provide culturally sensitive care to refugees	Not at all, Sometimes, Usually, Always

In order to reduce selection bias, we included schools whose response rates were above 25%. Two schools did not meet this threshold and were omitted from the final analysis. Restrictions on student e-mail survey distribution at these schools led to inconsistent dissemination compared to the other ten schools, likely contributing to the lower response rate.

Statistical analyses were conducted using IBM SPSS Version 24. Descriptive statistics are reported. Pearson’s correlation was used for subanalysis of the association between impact of GH opportunities on residency selection and level of desire to work with underserved populations in future career.

## Results

### Participants

Seven hundred and seven students completed the survey out of a total of 1554 possible students from the 10 included schools (46% response rate) (Table 2).

**Table 2:** Participant Chosen Field of Residency and Current Institution

**Chosen Field of Residency (N = 707)**	**N**	**% of All Respondents**
Internal Medicine	118	17%
Surgery	81	12%
Emergency Medicine	80	11%
Pediatrics	64	9%
Family Medicine	64	9%
Obstetrics & Gynecology	53	7%
Psychiatry	50	7%
Anesthesiology	39	6%
Radiology	21	3%
Neurology	20	3%
Otolaryngology	16	2%
Ophthalmology	16	2%
Med-Peds	14	2%
Dermatology	14	2%
Orthopedics	14	2%
Urology	13	2%
Physical Medicine & Rehabilitation	10	1%
Pathology	10	1%
Radiation Oncology	6	1%
Other	4	1%
**Institution (N = 707)**	**N**	**% of All Respondents**
Medical College of Wisconsin	95	13%
Case Western Reserve University School of Medicine		
University Program College Program	79 21	11% 3%
Loyola University Chicago Stritch School of Medicine	78	11%
University of Utah School of Medicine	76	11%
University of Wisconsin School of Medicine and Public Health	76	11%
University of Illinois at Chicago College of Medicine	74	11%
University of Cincinnati College of Medicine	71	10%
Wright State University Boonshoft School of Medicine	67	9%
Northwestern University Feinberg School of Medicine	46	7%
Mayo Clinic Alix School of Medicine	24	3%

### Influence of residency choice

Thirty-three percent of students rated the availability of GH experiences during residency as “moderately” or “very important” when selecting a residency program. Regarding formal GH training opportunities, 26% of students reported it as “moderately” or “very important” that a residency program has an established GH curriculum (track, pathway, certificate, or group). Higher proportions of respondents ranked GH experiences as important in residency selection among those applying to Internal Medicine/Pediatrics (8/14, 57%), Otolaryngology (8/14, 50%), Obstetrics and Gynecology (26/53, 49%), Ophthalmology (7/16, 44%), Dermatology (6/14, 43%), and Pediatrics (26/64, 41%) (Table 3). Eleven percent of students endorsed that they would only consider residencies that offered GH experiences. The most common reasons for desiring GH experiences in residency included the opportunity to work in low-resource settings (343/447, 77%), ability to learn about new cultures (282/447, 63%), chance to travel (276/447, 62%), and interest in working internationally for at least part of their career (258/447, 58%).

**Table 3:** Influence of Global Health on Residency Selection (N = 707)

	**N**	**%**
When you are selecting your residency program, how important to you is the potential for Global Health **opportunities** at the institution?		
Not important	260	37%
Slightly important	215	30%
Moderately important	161	23%
Very important	71	10%
How important is it to you that your residency program has an **established Global Health curriculum** (track, certificate, or group)?		
Not important	298	42%
Slightly important	225	32%
Moderately important	151	21%
Very important	33	5%
I will only consider residencies that offer Global Health opportunities.		
Strongly disagree	385	54%
Somewhat disagree	163	23%
Neither agree nor disagree	81	12%
Somewhat agree	59	8%
Strongly agree	19	3%
Reasons for desiring global health opportunities in residency – Multiselect Item (N = 447)		
Opportunity to work in low-resource settings	343	77%
Opportunity to learn about new cultures	282	63%
Opportunity to travel	276	62%
Interest in working internationally for at least part of my career	258	58%
Desire to learn about medical diagnoses not common in the U.S.	219	49%
Opportunity to work on foreign language skills	193	43%
Sense that a GH experience will improve my compassion and empathy	182	41%
Research interests abroad	85	19%
Other reason for desiring GH opportunities	13	3%
Students who rated Global Health opportunities as a moderately or very important factor when selecting a residency program (N = 707)		
**Residency Field Applied To**	**N/Total from Residency Field**	**%**
Med-Peds	8/14	57%
Otolaryngology	8/16	50%
Obstetrics & Gynecology	26/53	49%
Ophthalmology	7/16	44%
Dermatology	6/14	43%
Pediatrics	26/64	41%
Family Medicine	24/64	38%
Emergency Medicine	28/80	35%
Radiation Oncology	2/6	33%
Surgery	27/81	33%
Urology	4/13	31%
Physical Medicine & Rehabilitation	3/10	30%
Anesthesiology	10/39	26%
Neurology	5/20	25%
Other	1/4	25%
Psychiatry	12/50	24%
Internal Medicine	28/118	24%
Radiology	4/21	19%
Orthopedics	2/14	14%
Pathology	1/10	10%
**Total**	**232/707**	**33%**

### Career plans

After training, 65% of students envision practicing internationally in some form. Ten percent plan to spend a significant amount of time practicing internationally, while 55% anticipate a minority of their time spent practicing internationally. Finally, 84% of students plan to work with underserved populations in some capacity, with 50% of students stating that they would like to primarily work with underserved populations. Of the 232 students who rated GH experiences in residency as “moderately” or “very important,” 70% plan to work primarily with underserved patients in their careers, compared to only 38% among students who rated GH experiences as “not important.” Medical students’ interest in GH experiences and desire to work with underserved populations were significantly correlated (R = .326, *p* < 0.001) (Table 4).

**Table 4:** Future Career Plans

	N	%
In an ideal world, the location of my future practice would be:		
Completely in the U.S.	245	35%
Primarily in the U.S. with minor portion internationally	391	55%
Majority in the U.S. with a significant portion internationally	60	8%
Majority internationally	11	2%
When I have completed medical training, I hope to work with:		
No specific interest in working with underserved populations	115	16%
A minor portion of my patients who are underserved	221	31%
Primarily underserved populations	356	51%
Only underserved populations	15	2%

### Prior GH experience

Two-hundred and twenty students (31%) have participated in an elective rotation in a limited-resource setting, either globally or locally. Students participated in a wide array of experiences, including international (76%), local low-resource settings (46%), immigrant health (12%), refugee health (9%), Indian Health Services (4%), and US-Mexico border (2%). The large majority (78%) of students who have completed one of these experiences in medical school state that they are more likely to seek similar GH experiences in residency as a result of this elective experience. For students who did not complete a GH elective, cost was the most common barrier (52%) (Table 5).

**Table 5:** Global Health Experiences during Medical School

	N	%
Did you participate in an elective rotation in a low-resource setting (e.g. internationally or Indian Health Services)?		
Yes	220	31%
No	487	69%
If yes, how did this experience affect your likeliness to seek further global health experiences during residency?		
Slightly less likely	2	1%
Neutral	46	21%
Slightly more likely	87	40%
Much more likely	83	38%
Global Health Elective Settings - Multi-select item		
International	168	76%
Local low-resource setting	101	46%
Immigrants	27	12%
Refugees	20	9%
Indian Health Services	8	4%
Other GH elective	5	2%
US-Mexico border	4	2%
Reasons for Not Doing a Global Health Elective - Multi-select item		
Cost	254	52%
I did not have interest in doing so	181	37%
Inability to secure time for the rotation	172	35%
Family obligations	88	18%
Other reason for no GH elective	43	9%
Not available at my institution	25	5%
Concern for my own safety	22	5%
Concern for my own health	19	4%

## Discussion

In accordance with previous studies, our data demonstrates that the opportunity for GH experiences is an important factor for medical students when selecting a residency program [1, 2, 14–16]. One-third of medical students surveyed in our study endorse the importance of GH opportunities in their decision on where to apply for residency. Over a quarter of applicants are also seeking a formal GH curriculum when evaluating residency programs, and one in nine are only considering programs that have GH opportunities. Nearly a third of fourth-year students have participated in a GH elective during their training and the majority of these are more likely to seek similar experiences in residency as a result. Cost was a barrier to participating in GH experiences in over half of students, but the COVID-19 pandemic has resulted in a surge of virtual innovation to regain and strengthen GH partnerships, which may remove this barrier and allow for more involvement of trainees. The recruitment of applicants is important to every institution and offering GH electives can be a valuable recruitment tool.

Given the educational benefits and expanding interest in GH training [1, 7], providing quality GH education and experiences during residency is not only important for resident instruction and training, but can give training programs a competitive recruitment advantage. Our findings concur with other studies that have demonstrated the importance of GH in residency selection, though ours is unique in that it explores perspectives from the largest group of medical students on this topic to date. A survey of graduates of a family medicine residency program showed that the presence of an international health track was the factor that most positively influenced their choice of residency training site over others. Additionally, graduates who participated in the international health track were more likely to have come a greater median distance from their medical school or home, suggesting that the opportunities in global health widened the geographic recruiting scope for their residency program [14]. In a study surveying first-year emergency medicine residents, 62% of respondents who interviewed at programs with international opportunities rated this as a positive factor when ranking programs [15]. In a survey of graduating pediatric residents in 2008, 22% of respondents rated GH training as an “essential/very important” factor in selecting a residency program [1]. A survey of ophthalmology residency applicants showed 95.4% of respondents had interest in participating in a GH experience during residency with 52.1% being “extremely interested”. Indeed, the availability of GH experiences during residency increased an applicant’s interest in a program for two-thirds of individuals [16].

Our data correlates with studies published by Thompson et al and Camicci et al which demonstrate that students who are interested in GH opportunities during residency are more interested in working with underserved populations in their future careers [2, 16]. With this growing interest in providing care in limited-resource settings, residencies should consider development of formal GH curricula to ensure proper preparation and training for these situations. Further, a large majority of students in our study report plans to practice for a portion of their career internationally, including students who did not rate GH experiences as important in their residency choice. It is interesting that one third of respondents think that GH experiences in residency are important, while two thirds plan to work internationally in some form after graduation. Our survey did not determine a reason for this difference, though this does support the conclusion that there is a strong interest in GH among medical trainees and that residencies should provide GH education to ensure they are adequately prepared for their future careers.

### Limitations

This study has several limitations, including the total response rate of 46%. A total of ten different medical schools and 707 respondents were included. While the schools surveyed were limited to the Midwest, this is a substantially larger survey of medical students than in any other study focusing on GH training in residency. An additional limitation of this study is that the psychometric properties of the survey developed have not yet been determined. Using the results of the smaller pilot survey, items were determined by author expertise and consensus.

## Conclusion

Our world is continuing to shrink as a result of technologic advances, ease of travel, and human migration. In this reality, physicians need the skills necessary to provide high quality medical care for patients across varying cultures and backgrounds. GH education and experiences have shown great value in medical training with multiple studies revealing that these experiences provide unique and effective opportunities for clinical and personal growth that strengthen all six Accreditation Council for Graduate Medical Education (ACGME) core competencies [4]. This study demonstrates that the opportunity for GH experiences is an important factor for students when considering residency choice. Trainees who are interested in GH experiences in residency are more interested in working with underserved populations in their future careers. Therefore, by investing in GH education and experiences during residency, institutions can equip their residents to be effective and adaptable clinicians in multiple clinical settings, both locally and globally, and enhance the attractiveness of their programs to prospective trainees and applicants.

## Data Availability

The datasets during and/or analyzed during the current study available from the corresponding author on reasonable request.

## Abbreviations

GH: Global Health
US: United States
ACGME: Accreditation Council for Graduate Medical Education
